# Rwanda's Resiliency During the Coronavirus Disease Pandemic

**DOI:** 10.3389/fpsyt.2021.589526

**Published:** 2022-01-27

**Authors:** Elizabeth Farrah Louis, Dominique Eugene, Willy Chrysostome Ingabire, Sandra Isano, Judite Blanc

**Affiliations:** ^1^Fogarty International Center, Harvard University, Boston, MA, United States; ^2^Department of Psychiatry, Boston Medical Center, Boston University School of Medicine, Boston, MA, United States; ^3^Institute for Social Innovation Fellow, Fielding Graduate University, Santa Barbara, CA, United States; ^4^University of Global Health Equity, Kigali, Rwanda; ^5^Department of Psychiatry & Behavioral Sciences, Center of Translational Sleep and Circadian Sciences (TSCS), University of Miami Miller School of Medicine, Miami, FL, United States

**Keywords:** Rwanda, resilience, COVID-19, mental health, public health system

## Abstract

The coronavirus disease (COVID-19) pandemic has illustrated the wide range of preventative measures and responsive strategies of low- and middle-income countries (LMICs). LMICs have implemented lessons learned from previous periods of epidemics and uncertainties. Rwanda's pre-existing decentralized healthcare and mental health system which are in response to the mental health distress from the 1994 genocide, continues to be a formidable system that collaborate and combine efforts to address people's mental health needs. COVID-19 has heightened or exacerbated people's mental health within the country. Rwandans have been exposed to and endured adversities, yet their cultural forms of resilience serve as a mental health protective factor to also overcome COVID-19. Nonetheless, Rwanda has engaged in interventions targeting public safety, social and economic protection that specifically address vulnerable communitie's mental health needs. Lessons from preparedness for the Ebola virus disease (EVD) epidemic has contributed to Rwanda's organization and approach to combating COVID-19. Policies and best practices that were enacted during the EVD outbreak have guided Rwanda's response within the healthcare and mental health system. Coincidentally, this outbreak emerged during the 26th commemoration of the 1994 genocide against the Tutsi. Although for the first-time post genocide, Rwanda was not able to engage in public traditional forms of collective mourning and community healing, evidence of Rwandan's resilient spirit is demonstrated. Community resilience has been defined by Magis [401] as the “existence, development and engagement of community resources by community members to thrive in an environment characterized by change, uncertainty, unpredictability and surprise.”. Referring to this definition, community resilience has been an interwoven into the cultural framework that guided Rwandans in past challenges and continues to be evident now. Rwanda's resilience throughout this pandemic remains through ongoing psychoeducation, community awareness of mental health concerns, collective messages of highlighting mental health support, and solidarity. The global community can gain knowledge from Rwanda's learned lessons of their past which has positioned itself to stand on its resilient values in times of uncertainty such as COVID-19 and endeavor to overcome through national cohesion.

## Background of Rwanda

Known as the “land of a thousand hills,” Rwanda is a low-income mountainous country located in East Central Africa. It is surrounded by the Democratic Republic of Congo, Tanzania, Burundi, and Uganda. Rwanda's population is estimated at approximately 12,374,397 people (1). Thomson (2) notes that tourists find modern Rwanda to be an attractive, peaceful travel destination. Nevertheless, modern day Rwanda was not always the ideal place to visit. Prior to 1994, nearly half a million Rwandans (particularly the Tutsi group and some Hutus) were decimated. In the late 1980's, Rwanda's health quality and longevity had the lowest life expectancy amongst all countries (3). By mid-1990's, the genocide contributed to the increase of infectious disease and unsafe births (3). As the country attempted to rebuild its health infrastructure in the 2000's, the country became plagued with human immunodeficiency virus (HIV) and acquired immunodeficiency syndrome (AIDS) (3). Thus, in this article, we attempt to dissect the historical and psychosocial manufacture behind Rwanda's spectacular transformation in 25 years, and how lessons learned from past disasters and health crises are leveraged during this current coronavirus disease 2019 (COVID-19) pandemic period. We posit that there are some cultural values specific to the Rwandan people that serve as anchors for such transformation, that we perceive as the Rwandan resilience.

## Public Health System and Mental Health Integration

The history of Rwanda's psychiatric system can be divided in four major periods: 1.) The pre-colonial period, when the only mental healthcare available was based on traditional practices. 2) The colonial period, marked by the inputs of Western medicine 3) The post-independence period, that started from 1962, characterized by the construction of the first psychiatric hospital of the country; Neuro-Psychiatric Hospital Caraes Ndera, and the fourth stage which is the post-genocide period, which begun following the 1994 events (4).

During the pre-colonial period, traditional practices were the earliest forms of treatment for mental health. Traditional practices were a form of healing that originated from early Rwandan culture to address the “ancestral spirit of a deceased family member who was unhappy or angry” (5). Mental health treatment involved a diviner or traditional healer who intervened to address the unhappy or angry spirit which could be an ancestor from family lineage (5). Within the Rwandan context, there are different treatment options such as the “ritual of Kubandwa and Guterekera, which paid homage to certain spirits” (p.4). For example, one of these rituals paid their respects to Ryangombe, a divinity, representing the source of love, peace, and fertility (6). These indigenous healing practices were widespread throughout Africa and efforts were made to realign broken ancestral harmony that caused mental illness (5). Moreover, protection was a common belief that centered around God and ancestral spirits to protect individuals and their families. This protection extended to physical and mental health, which Rwandans believed if compliance with the “ritual obligations to honor the perceived protector, or from the ill will of an enemy who is jealous or who wants revenge” then reconciliation between God/ancestral spirits (the protector) and the person experiencing mental health issues was necessary [(5), p. 4].

Unfortunately, during colonial rule, traditional practices to treat mental health were considered pagan and denounced by Western religions. Under Belgian colonization, starting in 1922, Western approaches of medical care entered Rwanda through missionaries. Since there were no mental health facilities available to treat Rwandans, they were sent to Bujumbura, Burundi for care (5). When Rwanda gained its independence in 1963, these psychiatric patients were sent back to Rwanda and were either welcomed by family or sent to the Central Prison of Kigali. Before the 1994 genocide, there was only one hospital for mental health disorders, Caraes Ndera Neuropsychiatric Hospital. The hospital was founded in 1968 by the “Brothers of Charity,” which is a pontifical religious organization, in response to the government's request to receive mental health patients held in prisons across the country (7). As written by Brother Dr. Rene Stockman (8), the patients who were once chained like animals received proper care and treatment upon admission to Caraes Ndera, which improved their conditions (8).

The healthcare system was one of the sectors most affected by the 1994 genocide in Rwanda (9). Some health care providers were killed while others fled the country (9). During this period, infectious diseases such HIV and tuberculosis were on the rise; the life expectancy was the lowest in the world, and the country had no budget to respond immediately (9). The rate of post-traumatic stress disorder (PTSD) in the general population was 28, and 54% of those with PTSD suffered from major depression as a comorbidity (10).

Currently, the Ministry of Health (MOH) recognizes traditional medicine and is aware of the continuous practices of traditional forms of health alone or combined with modern medicine. MOH engages in ongoing efforts to involve indigenous practices in concert with the public health system (5). One of the government goals is to increase mental healthcare at the community level by the year 2024 (11).

The World Health Organization (WHO) defines integrated health services as “the management and delivery of health services so that clients receive a continuum of preventive and curative services, according to their needs over time and across different levels of the health system” (12). Dr. Brock Chisholm, who was the first Director-General of the WHO, said that “without mental health there could be no true physical health” (13). Today, evidence shows that patients with non-communicable diseases experience mental health problems and that having mental health problems increase the risks of developing non-communicable diseases such as heart disease as compared to normal population (13). The clear link between health and mental health is a fundamental concept when it comes to the integration of public health services to address overall wellness.

The Government of Rwanda's (GoR) 2018-2024 strategic plan for its health sector encompasses its decentralized healthcare (14). At the national level, there is the MOH, Rwanda Biomedical Center (RBC), and the National referral and University teaching hospitals (NRH/UTH). The MOH sets policies that mobilize resources, and ensure capacity of the healthcare personnel while RBC ensures the implementation of the health policies in an innovative way (14). There are provincial and referral hospitals that provide treatment of complex cases such as advanced life and trauma support, emergencies, intensive care, and psychiatric services. The lower levels are district hospitals, health centers, health posts, and community health workers as the entry point. The health centers provide promotional, preventive, and curative care and supervise health posts. Health centers provide services including psychosocial support, normal deliveries, minor surgeries, lab testing, and outreach activities such as vaccinations. Health centers refer to the district hospitals (14). Health posts at the cell level provide services including health education, child immunization, antenatal care, family planning, outpatient services and refer to the health centers (14). Both health centers and health posts constitute the primary healthcare which is usually the first contact patients have to address their mental health needs.

Despite the constraints of resources in terms of trained staff, proper equipment, and infrastructure, the GoR's decision to integrate mental healthcare in the decentralized public healthcare system is evidence across all levels from health posts to hospital settings (11). For example, each district hospital has a mental health department with psychiatric nurses and a clinical psychologist who provide outpatient and inpatient care including diagnosis, treatment, and counseling services. The mental health departments refer complicated cases to Ndera, the only national referral hospital for neuropsychiatric services in the country. Some documented examples of the utility of this integrated model depict an interdisciplinary approach. According to MOH, in 2009, there were 83,633 people who consulted mental health facilities including hospitals and psychosocial consultation services while 5,518 people were hospitalized for mental health disorders (11).

## Rwanda's Cultural Resilience

According to the former Rwandan Prime Minister, Dr. Pierre-Damien Habumuremyi (15), Rwandans are unique in the way they think, feel and act. It was these characteristics that were salient among Rwandans after the 1994 genocide. These Rwandan cultural values generate a community capable of adapting to disasters and finding common local solutions before resorting to outside support. Habumuremyi (15) describes four local cultural values that have contributed to Rwanda's achievements over the past two decades, namely: 1) a high level of patriotism and attachment to the homeland; 2) a permanent quest for success and self-sacrifice; 3) a high level of discipline and integrity and; 4) a sense of cooperation and mutual aid.

Habumuremyi (15) indicated that these four Rwandan cultural values have been leveraged by post-genocide leaders such as President Paul Kagame to involve the population in the process of socio-political and economic transformation of the country. These cultural values are representative during COVID-19 through the efforts of the government and the local people to support one another; it is evident in the promotion of resilient mental health and reliance on pre-existing mental health and healthcare resources while adapting to unprecedented circumstances (16). These virtues that originate from the Rwandan culture can be seen through concerted efforts of mobilization of the people to engage in their community development (e.g., umuganda), maintain solidarity, and embrace the spirit of volunteerism (e.g., CHWs) (15, 16). For example, umuganda is a dedicated monthly Saturday where Rwandans from all over the country come together to serve their communities by working on a project to resolve a community issue or bolster efforts to increase environmental sustainability. The maintenance of solidarity is depicted through ongoing community led initiatives to make collective decisions about ways to govern local and national entities and using their nationality to rally around coming together to play a role in combatting national concerns such as COVID-19. In terms of the spirit of volunteerism, community health workers have contributed as the voice of the people to connect Rwandans to health and mental health services all within a volunteer model.

The Rwandan spirit encompasses one in which they believe that their country “belongs to all Rwandans irrespective of their differences in identity and ideology” (15). This form of national unity and social equity is displayed during the pandemic and illustrates how this way of living manifests throughout government and community levels with critical consciousness and modifications of practices to serve vulnerable populations while accompanying others during this difficult time. Through learned lessons of the past, Rwanda has positioned itself to stand on its resilient values in times of uncertainty such as COVID-19 and overcome through national cohesion.

## Lessons Learned From Ebola While Managing COVID-19

Rwanda is no stranger to devastating events that have occurred in the country. Rwanda lived through an important period of uncertainty between 2012 and 2014 and from 2018 to present when the Ebola virus disease (EVD) threatened to spill over from the neighboring Democratic Republic of the Congo and Uganda. EVD is a rare but severe, often fatal disease that has human-to-human transmission, with an average fatality rate of 50% (17–20). The Ebola epidemic exposed the level of inadequacies that existed amongst institutions that were set up to protect the public such as human resource shortages and inadequate financing (18).

In the United States, Rwandans became the target of rampant discrimination which consisted of school officials banning students from attending classes; suspension of travels to and from the country; travel visas being denied; and imposing 21 days of quarantine despite the long distance of 1,700 miles between Rwanda and the affected regions (21). The EVD epidemic raised questions about the fragility of the global system's ability to respond to those types of outbreaks and how confidence needed to be rebuilt in order to prevent future crises (18, 21).

The Ebola crisis drew attention to the importance of reducing infectious diseases that pose threats across national borders, especially individual health security (17). Rwanda had to implement precautionary measures to prevent the emergence of this virus. They equipped EVD prone border areas with emergency holding rooms, installed hand washing stations, and utilized EVD camera detections (22) to bolster safety. The Rwandan government trained doctors to handle EVD cases, provided vaccinations in communities as well as ensured that healthcare workers were safe by having designated specialized ambulances for transporting patients (22). With these strategies in place, Rwanda demonstrated its readiness to respond to any type of outbreaks, including COVID-19.

Experts working for the WHO in Rwanda witnessed how the country was able to use its skills and expertise from tackling EVD to manage the COVID-19 outbreak (23). Techniques used in preventing EVD in 2018 have been found to be useful now with this pandemic (23). Designated COVID-19 centers and screening mechanisms have been set up around borders to limit the spread of COVID-19. Measures that were implemented from the Ebola epidemic resurfaced to raise COVID-19 awareness (e.g., communication through radio, television, community leaders, community health workers (CHWs), health facilities, and social media) (23). The Rwandan government expanded its COVID-19 surveillance and testing facilities, a testament of the country's success with reducing community transmission; while healthcare personnel have been active in tracing potential sources of transmission (23). Rwanda also utilized rapid screening and isolation of people who tested positive for COVID-19 with the “RT-PCR (reverse transcription polymerase chain reaction) tests which are accurate” [26, p. 1]. Mutesa et al. (24) developed “an algorithm for pooling subsamples based on geometry that, at low prevalence, uniquely identifies infected individuals in a small number of tests.” By using their methods, the costs of mass testing are reduced while being able to quickly identify and quarantine individuals which in turn decrease the prevalence of COVID-19.

The MOH utilized pre-emptive measures during the Ebola crisis to respond to the coronavirus by quickly implementing contact tracing protocols (23). Rwandans and donors (e.g., C & D Pink Mange and the Jack Ma Foundation) from various companies mobilized to provide donations of over 25,000 test kits, 100,000 masks, 1,000 medical protective suits and face shields during the early stage of the pandemic (22). Rwanda has the capacity to test over 200 cases in approximately 4 hours for suspected cases to know their results (25). To date, Rwanda has the capacity to conduct a little over 5,534 tests a day and have tested over 255,959 people as of July 30, 2020 (16). Rwanda turned to digitized contact tracing by “using mobile phone data profiles to trace people who had contact with COVID-19 patients,” which has been effective (22). There are designated facilities where COVID-19 cases go to receive services with follow-up by emergency workers, often through home visits (16). Additionally, the Butaro Cancer Center for Excellence, provides additional support for patients who need cancer treatment by providing transportation to areas around the country (23). Healthcare providers work with CHWs to identify community members with health and mental health needs and share messages of COVID-19 (16).

In 2013, Rwanda pioneered the deployment of disease-specific funding to build its primary health care system, and it has maximized synergies between U.S. President's Emergency Plan for AIDS Relief (PEPFAR) and other bilateral, multilateral, and country health initiatives (3). Since then, Rwanda has improved the country's health services. Rwanda has come a long way in its ability to effectively care for its citizens.

## The Impact of COVID-19 and Rwanda's Response

The novel coronavirus outbreak was first declared a pandemic on March 11, 2020, by the World Health Organization. In Rwanda, the first case of COVID-19 was declared by the MOH on March 14, 2020. Similarly, to many other countries, the GoR took robust mitigation strategies that initially included the country-wide lockdown, which was later substituted by wearing of masks outside and a 9 pm to 5 am curfew (26). From the Gelfand et al. (27), the strict measures that Rwanda took can be characterized as a country of “cultural tightness,” a theory which refers to the level of “strict norms and punishments for deviance” (p. 2). Rwanda's efforts in combating COVID-19 included police patrol of neighborhoods after curfew and giving fines, reprimanding people who did not follow COVID-19 rules such as wearing a mask and having them stay in a stadium for further questioning and punishment. From Gelfand et al. (27), study, nations with higher levels of cultural tightness had lesser COVID-19 cases and deaths due to these nations prompt and proactive response to this pandemic and contributed to collective cooperation as an advantage to higher survival rates during a “collective threat” (p. 2). Globally, the COVID-19 pandemic has been far more than a health crisis: it has negatively impacted societies and economies in many spheres. In Rwanda, COVID-19 has caused many disruptions related to socio-economic factors including but not limited to unemployment, limited physical contact with family members, isolation of contacts and cases of COVID-19, social distancing, non-participation in care of sick loved ones, non-participation in the burial of loved ones who die, loss of productive livelihood, and stigma and discrimination of survivors (12).

There were many social aspects that were affected by the pandemic as people tried to adhere to policies to remain safe. Yet, people found it difficult, especially those from low-income settings who had limited access to personal protective gear and faced compounded challenges. For example, people lost their jobs and had reduced work hours. Due to the demand for goods and services, prices increased, there was a scarcity of familiar items, and people had to contend with the restrictions of movement (16). For others, the elimination of public transportation further created a strain on vulnerable populations. To address some of these challenges, the GoR, applied alternatives such as providing medical officials the ability to transfer patients to health facilities in different provinces (16). Also, a social support plan was developed to alleviate food insecurity in which food was delivered to some poor households in cities and rural areas (19). The Rwandan government's reliance on emergency food shortage reserves and loans from the World Bank and local Rwandan's collaboration in donating food while farmers continued to prepare for the agricultural season demonstrated the commitment to serve those in need and the country.

COVID-19 imposed a lockdown, which in turn imposed new ways of living. Most people shared a feeling of uncertainty about the future, as a result some people responded with fear and anxiety (26). Through different technological means of communication such as cell phones, radio and television, and social media platforms, people have adopted the coping strategies most applicable to them including talking to their relatives and friends, praying or attending their online church services (26). During the COVID-19 pandemic, through its national health implementation agency, RBC, and in collaboration with mental health patients, community health workers, healthcare providers, and partner organizations put in place guidelines to ensure continuous provision of mental healthcare at all levels including at the community and health facility levels (26). There are guidelines for specific groups including education for the general public; debriefing meetings for healthcare providers; recommendations on how to validate and support children and adolescents; support, inform, and prepare for elderly people; and engage, prevent, and address the barriers for people with disabilities; psychoeducation and mental health resources (26). Rwanda's response to the needs of its people intersected across existing multilateral systems with the involvement of stakeholders to alleviate the impact of this pandemic.

## COVID-19 Coincide With The 26th Commemoration

Rwanda's integrated mental and healthcare system was tested not only by COVID-19 but also by the 26th commemoration of the 1994 genocide against the Tutsi group. Historically, three ethnic groups: Twa, Tutsi, and Hutu comprised Rwanda's culture with a shared language. The Twa, who are the minorities, had no political voice in creating the country's infrastructure. During April 1994, Rwanda experienced genocide in which approximately one million people from the Tutsi ethnic and moderate Hutu were killed; 250,000 women were raped; and millions of Rwandans were displaced, or fled to other East African countries including Uganda, Congo, Kenya, and Tanzania (28). Studies conducted in the years following the genocide demonstrated that genocide survivors suffered from high rates of PTSD from witnessing violence and the killings of people with a machete (11, 29). In a study in 2012, it found that more than 26.1% Rwandan adults were diagnosed with PTSD and the majority of them had comorbid psychiatric disorders such as major depression (68.4%), substance dependence (7.6%), or somatic symptoms such as back pain (74.1%), and headache (71.2%)” (10). It is also important to note that epilepsy has been reported to increase as a consequence of the genocide, representing about 4.9% of the Rwandan population which further creates stigma (30).

The prevalence of PTSD, depression, trauma, and anxiety remains for both genocide survivors and perpetrators, with recurrent flare ups during the yearly genocide commemoration period. The commemoration is usually a seven-day period in April during which Rwandans gather to mourn the victims of the genocide. During this commemoration period, people living with chronic PTSD experience acute PTSD exacerbations characterized by flashbacks, agitation, self-mutilation, and anger, among other symptoms (29). Addiiotnally, substance abuse has increased in the aftermath of the genocide, widows and orphans are left to endure poverty and have limited access to education, while women were also victims of sexual violence (5). However, the country's choice to stay together, to be one Rwanda, was a difficult choice that was felt by Rwandans and its leadership. Through pain and sacrifice, the government asked its people to be resilient in their collective journey of living beyond survival (31). From this tragedy, many initiatives spearheaded by the government and the people garnered spaces for Rwandans to heal, share their stories of survival, and imprint the spirit of resiliency and hope (32). Post genocide research have “characterized three culturally specific Rwandan concepts that related to resilience: kwihangana, an intrapsychic creative process of drawing strength from within the self in order to withstand suffering; kwongera kubaho, affirmation of the reestablishment of the existential conditions for being; and gukomeza ubuzima, the moving forward in life by accepting ongoing struggles and fighting for survival” [33, p. 413; 34]. These cultural forms of resilience are embedded in Rwandans and have fostered reconciliation and restorative justice (33). Kwihangana stands for someone to be patient and strong while her or she endure challenges and know that his or her suffering will not last forever. This was a salient resilient process that post-genocide survivors held onto and continue to use to guide them with current and future challenges. Kwongera kubaho refers to the individual and community process that embedded in cultural affirmation and reestablishing community and relational conditions of healing, forgiveness and trust post-genocide to recenter and value the Rwandans as individuals and as a people. As or gukomeza ubuzima, this particular attitude provides a framing of life that guides Rwandans to continue to move forward despite the ongoing circumstances and hardships that one may face in order to survive.

In Rwanda, as the outbreak of COVID-19 coincided with the annual commemoration, Rwandans were forced to pay their respects in their homes and cope with their trauma and reminders of the genocide. There were no night vigils, village level discussions, and the annual Walk to Remember that have traditionally marked Rwanda's genocide commemorations (32). Genocide commemoration related talks were streamed on local media channels and social media. There were also special messages related to commemoration by some key personalities on TV, radios, and other media outlets. Special government notices were shared throughout healthcare systems and with mental health professionals to be vigilant of the mental health needs of the population during this sensitive and challenging period. During this period, kwihangana, kwongera kubaho, and gukomeza ubuzima will continue to be cultural anchors of resilience as Rwandans come together to overcome another adversity.

## Mental Health Distress and Response

Consequently, the lockdown has tested people's mental health coping strategies with being alone or with family members who also have their own forms of distress (34). Whether through social media platforms, to conversations with family and friends, and the concerns of healthcare workers, people are anxious, stressed, fearful, and uncertain about their future (26). Concerns related to financial support, maintaining social connections, the risk of turning to substance use to cope remains salient risk factors have also been present during this time (26). Some have turned to religion/spirituality to cope either through prayer or listening to sermons (35). With being restricted to the confines of one's home, there has been a rise of domestic violence and family conflict during this pandemic. For example, in some cases child support has ceased because jobs have been halted or men are no longer helping teen mothers and children financially (36). Couples who were on the verge of finalizing their divorce have had to stay with each other while teen mothers have had to stay in the same home of men who abused them or impregnated them (36). This creates conflict and further puts these women and children at risk for additional physical, emotional, and verbal abuse. The Rwanda Investigation Bureau and local authorities have received hundreds of calls a day around concerns of gender-based violence and interpersonal conflict during the lockdown period of this pandemic (36). Women and children in abusive situations are further at risk when the context of poverty and substance abuse is present in the home which can increase perpetrator's aggressive behaviors (26). Efforts to use local authorities to monitor cases and use digital services have been implemented along with raising awareness through media outlets (19).

During this pandemic, as mental health professionals, we have been working with various colleagues to create a hotline dedicated to mental health concerns (34). From our perspectives, patients with mental health disorders, healthcare providers, organizations, and CHWs have been working together diligently to make sure there are limited interruptions in psychiatric support for patients. The national healthcare insurance, Mutelle remains operable and its continuation during this challenging time ensured Rwandans had access to mental health services. Dr. Louis contributed to the mental health guidelines of MOH to demonstrate the importance of validating people's emotions and experiences while offering strategies to cope with COVID-19 (26, 34). Through our experiences in this setting, we have witnessed our family members, friends, and colleagues engage in regular check-ins by phone as a means of bolstering morale.

According to Zraly et al. (33) “human resilience, the capacity to function or adapt during or following exposure to adversity, can function to protect mental health among violence-affected populations (p. 411). Rwandans have been exposed to and endured adversities, their cultural forms of resilience serve as a mental health protective factor to also overcome COVID-19. Additional forms of mental health support include patient access to discounted psychotropic medications, general and psychiatric nurses are trained to administer psychiatric medications, at each healthcare facility there is at least one mental health professional, and patients can engage in individual and group therapy. Contextual factors such as socio-economic status are taken into consideration (community-based health insurance known as “Mitiweri” Mutuelle de sante, whereby people pay for enrollment according to their social category; most poor being covered by the government); for more severe patient cases psychiatric inpatient care is available, and mental health professionals and CHWs collaborate to assist community members manage their mental distress (16).

While these are great initiatives, some challenges that still remain include stock out of psychiatric medications such as valproic acid for seizures and haloperidol for schizophrenia, reliance on CHWs to identify community members who need support, limited resources to address socio-economic issues, stigma of having a mental illness, limited mental health providers to address patient's needs in rural areas, high turnover of general and mental health nurses who make up a large portion of the health system, and limited resources to provide mental healthcare to children and persons with disabilities (16).

Recommendations of mental health strategies such as exercise, relaxation techniques, limiting intake of news on COVID-19, and speaking to a mental health provider appears to be helpful for some Rwandans (26). The reality for other Rwandans is that this unfortunate time causes additional stress, making it difficult for them to implement the aforementioned. In order to address these various levels of distress and reduce maladaptive coping, continued affirmation and validation of people's feelings, self-compassion, empathy, reinforcing resilient values, and normalizing experiences during this unprecedented time is vital to overcome this pandemic (34). The incorporation of cultural and community oriented mechanisms such as religion and spirituality and attending services, community meetings led by elders, youth led involvement in healing practices and conflict resolution, and umuganda (day of service) that already exist to address positive mental health is also needed especially since during the period of the pandemic, there can be some anxiety and fears of accessing care within a medical system. Rwandans are currently using their past experiences of coping with adversities to deal with the changes of normality due to COVID-19. The resilient principles of the Rwandan people can and will continue to provide them with guidance on how to overcome and rely on each other.

## Community Resilience Within The Rwandan Context

When it comes to the definition of community resilience, experts in disaster preparedness and response argued that a required paradigm shift and a new national culture of disaster resilience needs to occur. However, Patel et al. (37)'s review on the community resilience definition found no consensus on what such a culture would look like within our communities. Rather they noted that there is a wide range of components that have been proposed within the general notion of community resilience that are worthwhile to explore in the Rwandan context, namely they are: Local knowledge: factual knowledge acquired by the community as it relates to a disaster; community networks and relationships: community connectedness and cohesion; health: The pre-existing physical and mental health of a community and “delivery of health services after a disaster;” resources: tangible and intangible resources that are available and fairly distributed in the community; economic investment: post-disaster direct and indirect economic costs, assessment of community needs and potential for economic growth; preparedness: the active involvement at the individual, family, community and government levels to engage in planning, risk management and preparedness initiatives; mental outlook: meaning making, attitudes, feelings, and points of views after a disaster that affect a community's outlook through “hope, adaptability, ”and “acceptance of uncertainty and change” (p. 15).

For instance, through government and community-based approaches, factual knowledge, collective efficacy was emphasized to ensure that Rwandans had access to correct information and fostered the importance of discipline and integrity to combat COVID-19. Additionally, appropriate hand washing and other preventative mechanisms were provided through different outlets to promote education, empower Rwandans to care for their health and others, and the government in collaboration with national and international organizations collaborated with staff to train them on appropriate procedures during this pandemic. The use of many languages such as Kinyarwanda, French and English is useful in combination with the component of local knowledge and culture are also examples of community resilience in order to reduce negative outcomes and help the community understand its risks. While effective communication, including risk or crisis communication, have been found to be crucial tools for a community in addressing disaster outcomes.

Similarly, the positive impact of connectedness and cohesion within the element of community networks and relationships which aligns with Rwanda's patriotism and attachment to the homeland are believed to help people cope with uncertainty after a disaster. The decentralized health system that encompasses mental health services is undoubtedly important for communities to recover from disasters such as COVID-19. Understanding and addressing health and mental health vulnerabilities can build resilience before a disaster and mitigate long-term issues after a disaster which is evidenced throughout Rwanda's precolonial, colonial, post-independence and post-genocide periods. Moreover, within the framework of governance and leadership, ensuring the engagement of front-line leadership during crises such as Ebola and COVID-19 are clear at the local level and also represents community resilience.

An equitable distribution of resources is essential in the short term. From tangible supplies, such as food, psychosocial support, distribution of masks, the use of drones to deliver medicine to underserved communities, and providing free ongoing COVID-19 testing to the population are valuable resources that are associated with stronger resilience factors. Assessing a community's current economy and developing its ability to sustain economic growth were also noted as important areas of concentration after a disaster. The importance of preparedness across a number of levels, including the individual, family and government have often been acknowledged.

Last but not least, mental outlook which is different from mental health refers to attitudes, feelings and views when facing the uncertainty that usually occurs in the aftermath of a disaster. Thus, mental outlook is considered as the greatest resilience-promoting factor within a community through a focus on sub-components such as hope and adaptability. The mental outlook of a community is consequently determinant in the preparedness of affected individuals to continue on in the face of uncertainty (37). The mental health outlook for Rwandans as with previous periods of trauma, uncertainty and disasters have been to engage in collective cooperation and mutual aid, knowing that the journey for better days and success is achieved through unity and self-sacrifice with the hope of a better tomorrow for all Rwandans.

## Conclusion

Rwanda has continuously demonstrated its cultural forms of resilience to address periods of uncertainty that are interwoven by values that guide its beliefs and actions. COVID-19 is not an exception to this country's management of its preventative and responsive measures that have been applied in previous health emergencies such as Ebola. The decentralized healthcare system with the incorporation of a community based mental health approach provides essential services that further support Rwandans during a pandemic and through another commemoration that elicited mental health distress. Community resilience thrives in Rwanda and its principles are embedded at all levels and enrich the lives of its citizens to overcome challenges that are present and will arise in the future. This LMIC can teach the world about its evolving journey for progress, sustained collective unity, mental health priority, culturally informed resources, and community-oriented mechanisms that all contribute to Rwandan resilience.

## Data Availability Statement

The original contributions presented in the study are included in the article/supplementary material, further inquiries can be directed to the corresponding author/s.

## Author Contributions

EL, DE, WI, SI, and JB provided their contributions throughout this article and are in agreement to be accountable for the content of the work. All authors contributed to the article and approved the submitted version.

## Funding

EL and DE are supported by the Fogarty International Center and National Institute of Mental Health (No. D43TW010543). JB's work is funded by a grant from the National Heart, Lung, and Blood Institute and National Institutes of Health (Nos. T32HL129953 and 7R01HL142066-04). The content is solely the responsibility of the authors and does not necessarily represent the official views of the National Institutes of Health.

## Conflict of Interest

The authors declare that the research was conducted in the absence of any commercial or financial relationships that could be construed as a potential conflict of interest.

## Publisher's Note

All claims expressed in this article are solely those of the authors and do not necessarily represent those of their affiliated organizations, or those of the publisher, the editors and the reviewers. Any product that may be evaluated in this article, or claim that may be made by its manufacturer, is not guaranteed or endorsed by the publisher.
